# Bio-Inspired Riblet Structures on Hyperelastic FKM Sheets: A Simulation-Guided Process-Window Screening for Roll-to-Roll Hot Embossing

**DOI:** 10.3390/biomimetics11070510

**Published:** 2026-07-21

**Authors:** Jiangpeng Liu, Jie Xu, Chaogang Ding, Debin Shan, Bin Guo

**Affiliations:** National Key Laboratory for Precision Hot Processing of Metals, Harbin Institute of Technology, Harbin 150001, China; 21b909095@stu.hit.edu.cn (J.L.); shandb@hit.edu.cn (D.S.); guobin@hit.edu.cn (B.G.)

**Keywords:** shark-skin-inspired V-shaped riblets, roll-to-roll hot embossing, hyperelastic FKM, microstructure forming, retained morphology, finite-element process screening

## Abstract

V-shaped riblets are widely studied shark-skin-inspired microstructures, but their continuous high-fidelity replication on soft hyperelastic substrates remains challenging because large substrate deformation complicates complete profile filling. This study establishes an Abaqus-based finite-element process-window and morphology-screening method for roll-to-roll (R2R) hot embossing of 100 μm-scale V-shaped riblets on a fluoroelastomer (FKM) sheet as a model hyperelastic substrate. A Yeoh hyperelastic law calibrated from room-temperature uniaxial tension was implemented in a three-dimensional large-deformation contact simulation. Embossing temperature *T* and imposed nip-compression depth *D* were examined as screening variables, with formed riblet height, filling ratio, and auxiliary field indicators used to evaluate the forming response. The simulated filling ratio increased from about 69% to 76% as *T* increased from 120 to 180 °C and from about 39% to 76% as *D* increased from 60 to 120 μm, indicating that nip-compression depth exerted the stronger geometric control over profile filling. R2R hot embossing experiments and laser-confocal profilometry evaluated the retained riblet morphology. For the 160 °C *D*-series, the measured retained morphology followed the simulated filling trend, with a filling-ratio RMSE of 3.09 percentage points and top-width RMSE of 2.01 μm. The integrated numerical–experimental framework provides an experimentally supported manufacturing basis for process-window selection and retained-morphology control in the R2R hot embossing of riblet-textured hyperelastic sheets.

## 1. Introduction

Biomimetic surface engineering uses structural features found in nature as design templates for artificial materials and manufacturing processes. Among these templates, shark skin has motivated engineered riblet surfaces because its aligned denticle and ridge features provide a biological design source for simplified, controllable streamwise microgeometries [1,2,3].

Riblet surfaces constitute an established family of bio-inspired geometries, including blade-shaped, trapezoidal and triangular, or V-shaped profiles. V-shaped riblets have long served as well-established benchmark microstructures in riblet research [4,5,6,7,8,9,10]. Their height, width, and cross-sectional shape are clearly defined, and previous experimental and numerical studies show that riblet geometry and characteristic size are central to their potential hydrodynamic function. Accurate replication of a literature-grounded profile therefore constitutes an independent manufacturing problem alongside functional evaluation.

For practical use, riblet textures must be transferred from laboratory-scale rigid specimens to large-area surfaces. Many engineering surfaces relevant to drag reduction are curved, non-planar, or geometrically complex, so microstructured flexible sheets offer a practical route: they carry a designed microtexture while conforming to curved or complex substrates. This requirement makes high-fidelity replication of riblet microstructures on flexible or elastic polymer sheets a central manufacturing task [11,12,13].

Roll-to-roll hot embossing is a promising route for this purpose because it continuously replicates microstructures on polymer films and flexible substrates with higher throughput than batch plate-to-plate processes. Previous R2R hot embossing studies have demonstrated the value of continuous or semi-continuous replication for microstructured polymer films, including large-area patterned films and polymeric microstructures. These advantages make R2R hot embossing well suited to riblet-textured sheet preparation, where repeatability, area coverage, and process efficiency must be combined with local profile fidelity [14,15,16,17,18,19,20,21].

For elastic or hyperelastic sheets, R2R hot embossing of riblet structures introduces a forming problem governed by large deformation, contact-dependent riblet-profile filling, and post-embossing profile retention. Studies on fluoroelastomer (FKM) temperature-dependent behavior and elastomer-film hot embossing show that microstructure transfer to elastomeric materials requires the coordinated control of tool geometry, thermal softening, and profile retention [22,23,24,25,26]. For 100 μm-scale V-shaped riblets, temperature, nip-compression depth, and post-embossing profile retention must therefore be considered together.

Pure experimental trial-and-error is inefficient for this manufacturing problem because each R2R test reveals only the final measured morphology, while the respective influences of embossing temperature and nip-compression depth and the associated local contact, strain, and stress fields remain difficult to resolve separately. Finite-element simulation provides a useful way to organize process conditions before expanding experiments, especially for micro-embossing and roll-embossing problems in which filling and process variables are coupled [27,28,29]. A remaining need is therefore an integrated numerical–experimental framework that relates material behavior and forming-stage responses to the morphology retained after release and cooling.

To address this manufacturing problem, the present study takes the widely studied shark-skin-inspired V-shaped riblet as a representative microstructure and FKM as a representative hyperelastic substrate. An integrated numerical–experimental framework is developed to investigate the continuous replication of this microstructure by R2R hot embossing. Room-temperature tensile tests are used to calibrate the constitutive model, and finite-element simulations screen the effects of embossing temperature and nip-compression depth on the forming response. R2R experiments and laser-confocal measurements are then used to evaluate the replicated morphology. The framework provides a manufacturing basis for producing microstructured flexible elastomeric sheets with potential applicability to curved or geometrically complex surfaces.

## 2. Methods

**Figure 1:**
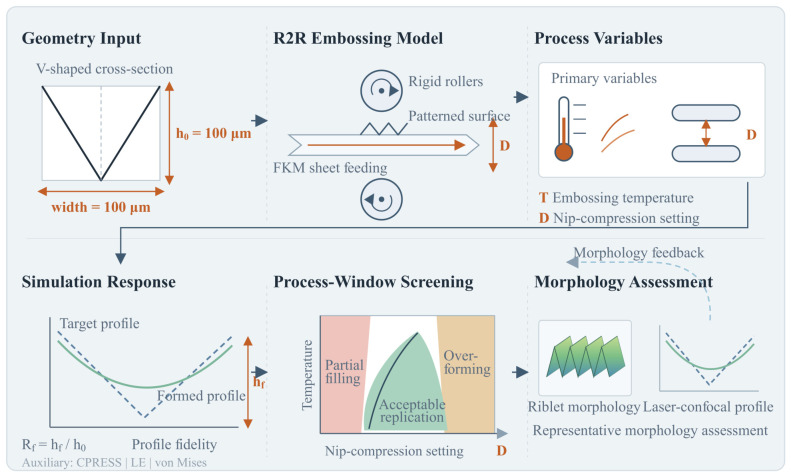
Integrated numerical–experimental workflow linking riblet geometry, R2R hot embossing simulation, process-condition screening, experimental fabrication, and morphology assessment. Solid arrows indicate the forward workflow, whereas the dashed blue arrow denotes morphology feedback. Orange annotations identify geometry and process variables; the blue dashed and green solid profiles denote the target and formed/assessed profiles, respectively; and the red, green, and orange shaded regions denote partial filling, acceptable replication, and over-forming.

The integrated numerical–experimental framework is summarized as a single workflow in Figure 1. The workflow begins with the definition of the target riblet geometry and calibration of the FKM constitutive response from room-temperature tensile tests. The calibrated material model is then implemented in a three-dimensional R2R hot embossing simulation, in which embossing temperature *T* and prescribed nip-compression depth *D* are screened through profile-filling and auxiliary-field responses. Candidate conditions identified numerically are subsequently examined through R2R hot embossing experiments, and the replicated morphology is characterized by laser-confocal profilometry. The workflow therefore connects material characterization, numerical process screening, experimental fabrication, and morphology assessment.

### 2.1. Material Characterization and Constitutive Model Development

**Figure 2:**
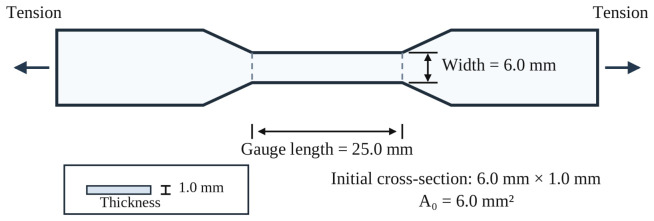
Tensile specimen.

Room-temperature uniaxial tensile data were used to establish the hyperelastic material input for the FKM sheet. The tensile response used for model fitting was obtained by averaging five replicate tests. The specimens had a gauge length of 25.0 mm, a width of 6.0 mm, and a thickness of 1.0 mm, corresponding to an initial cross-sectional area of 6.0 mm^2^. The specimen geometry is illustrated in Figure 2, and the averaged nominal stress–strain response was used for constitutive calibration.

Two hyperelastic descriptions were fitted to the averaged tensile response to select a stable material law for the subsequent large-deformation contact simulations. Incompressible Yeoh and second-order Ogden models were compared using Levenberg–Marquardt nonlinear least squares, with the objective function defined as the squared difference between experimental and model-predicted nominal stresses. The residual-change tolerance was set to 1 × $10^{-6}$, and the maximum iteration count was 10,000 [30,31]. The Yeoh strain-energy density adopted in the simulations was

(1) $$W=C_{10}\left({\bar{I}}_{1}-3\right)+C_{20}{\left({\bar{I}}_{1}-3\right)}^{2}+C_{30}{\left({\bar{I}}_{1}-3\right)}^{3}$$

The fitted Yeoh coefficients were used directly as the Abaqus material input, with a near-incompressible deviatoric response (ν = 0.49). The calibrated material input provides the room-temperature constitutive basis for the process-window screening simulations.

The fitted Yeoh constants used for the current Abaqus material input are summarized in Table 1.

**Figure 3:**
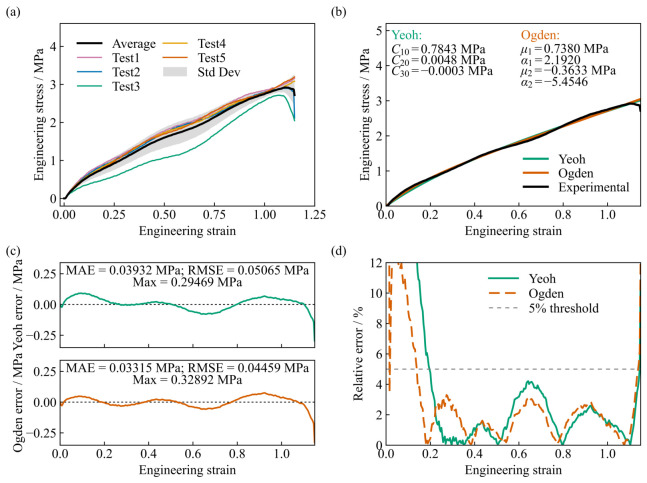
Room-temperature tensile characterization and hyperelastic fitting of the FKM sheet. (**a**) Averaged nominal stress–strain response with five replicate tests and standard-deviation band; (**b**) experimental response with the fitted Yeoh and Ogden curves and fitted parameters; (**c**) Yeoh and Ogden fitting residuals, where the green and orange curves denote the Yeoh and Ogden residuals, respectively, and the gray dashed lines indicate zero error; (**d**) relative-error comparison with the 5% threshold.

Figure 3a establishes the calibration response by showing the five tensile replicates, their average, and the standard-deviation band. The averaged response rises over most of the measured strain interval before the terminal stress drop, while the band captures the test-to-test dispersion used to assess the representativeness of the calibration data. In Figure 3b, both the Yeoh and second-order Ogden predictions closely follow the averaged experimental curve over most of the calibrated range, with the most visible departures concentrated near the terminal high-strain point.

The residual distributions in Figure 3c fluctuate around zero for both models. The Ogden fit gives the slightly higher $R^{2}$ and the lower MAE and RMSE, whereas the Yeoh fit gives the lower maximum absolute error, as quantified in Table 2. Figure 3d further shows that the relative errors remain below the 5% reference over most of the finite-stress calibration interval; the largest excursions are concentrated near the near-zero-stress origin and the terminal stress drop. Together, these panels distinguish the mean-error and endpoint-error characteristics of the two fits.

Both constitutive models reproduced the measured tensile response closely. As shown in Figure 3b and Table 2, the Yeoh fit contains a negative $C_{30}$ coefficient, whereas the Ogden fit contains negative $\mu_{2}$ and $\alpha_{2}$ parameters. For the Yeoh model, the strain-energy density and its derivative remained positive over the measured deformation interval. Considering its smaller parameter set and lower maximum absolute error, the Yeoh model was adopted for the subsequent simulations.

### 2.2. Geometry and Abaqus-Based R2R Hot Embossing Model

**Figure 4:**
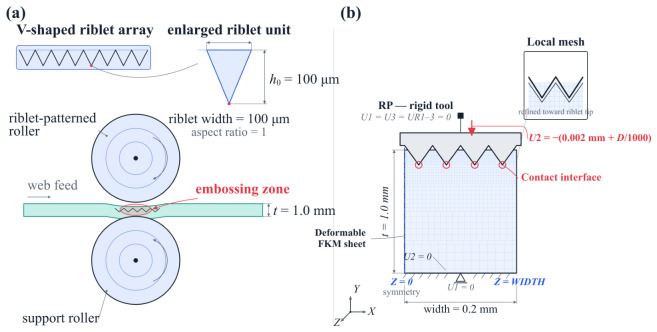
Finite-element representation of the local R2R hot embossing nip. (**a**) Roller–sheet configuration, material-transport direction, and nominal V-shaped riblet geometry; (**b**) local three-dimensional contact model showing the rigid-tool displacement, contact interfaces, sheet constraints, and graded mesh refinement.

The local model represents the embossing zone in which the FKM web passes between the forming roller and the support roller, as shown in Figure 4a. The forming surface carries periodic V-shaped ridges corresponding to a target riblet height $h_{0}$ of 100 μm, a nominal width of 100 μm, and an aspect ratio of 1. In the three-dimensional idealization, a 1.0 mm-thick deformable FKM sheet was placed beneath the rigid forming tool, and a local periodic geometry was retained to resolve riblet-profile filling at manageable computational cost.

Figure 4b translates the nip configuration into the local three-dimensional model and identifies the rigid-tool reference point, tool–sheet contact interface, sheet constraints, and refined forming region. Surface-to-surface penalty contact paired the rigid tool with the FKM top surface; hard pressure-overclosure was used in the normal direction and the baseline tangential response was frictionless. The sheet bottom was constrained in the vertical direction, and one bottom-center node was constrained to suppress in-plane rigid-body translation; symmetry was imposed at Z=0, and out-of-plane displacement was suppressed at the opposite width boundary. The rigid-tool reference point was constrained against lateral translation and rotation.

In the numerical model, *D* denotes the prescribed nominal nip-compression depth after the closure of the initial 2 μm tool–sheet clearance. It was imposed by translating the rigid forming tool downward by 0.002mm+D/1000, with *D* expressed in micrometers, as indicated by the displacement condition in Figure 4b. To evaluate the sensitivity of the local field response to tangential friction, an additional calculation at D=80μm used friction coefficients of 0.05 and 0.10 under the same geometric and thermal conditions as the frictionless baseline.

Table 3 summarizes this contact-sensitivity analysis. Introducing low to moderate tangential friction changed the maximum absolute logarithmic strain only slightly and kept ALLKE/ALLIE below 1.11 × $10^{-3}$, indicating a limited effect of tangential-contact choice on the main geometry-screening trends.

The temperature-series simulations used D=80μm as the fixed prescribed nip-compression depth to isolate the effect of embossing temperature. The *D*-series simulations used D=60, 90, 100, and 120μm under a fixed thermal context of 140 °C preheating and 160 °C embossing. The same displacement-controlled forming state and boundary conditions were applied throughout each series so that the effects of *T* and *D* could be evaluated independently within the local model.

Temperature *T* was introduced through an equivalent hot-state Yeoh-coefficient representation. The room-temperature Yeoh coefficients in Table 1 were used as the material input, and the hot-state coefficients were generated using a scalar temperature-softening coefficient s(T):

(2) $$\left(C_{10}\left(T\right),C_{20}\left(T\right),C_{30}\left(T\right)\right)=s\left(T\right)\left(C_{10,\mathrm{RT}},C_{20,\mathrm{RT}},C_{30,\mathrm{RT}}\right).$$

The scaling factors and corresponding hot-state coefficients used in the screening simulations are listed in Table 4. The 150 °C row was used for the contact-sensitivity calculation at D=80μm and was obtained by linear interpolation between the 140 and 160 °C scaling factors. This equivalent hot-state representation keeps the temperature-series and *D*-series comparisons on a common material-scaling basis.

The FKM sheet was discretized with 103,680 C3D8R reduced-integration elements and the rigid tool with 40 R3D4 elements, giving 103,720 elements and 118,810 nodes in total. Eight elements spanned the 0.2 mm out-of-plane width with dz = 0.025 mm. As shown by the local-mesh inset in Figure 4b, the mesh was refined progressively toward the riblet-forming contact region and graded into the bulk, with a minimum local element length of $h_{\min}$ = 2.5 μm.

The forming analysis used Abaqus/Explicit with nonlinear geometry, smooth displacement loading, and a mass-scaling target time increment of 1.0 × $10^{-7}$ s to accommodate large deformation and evolving contact [27,28,29]. At D=80μm, increasing the local in-plane mesh density from the reference discretization to its twofold refined counterpart changed both imprint depth and maximum displacement from 0.08200 to 0.08198 mm (approximately 0.02%). The kinetic-to-internal energy ratio remained below the 5% quasi-static criterion, with ALLKE/ALLIE = 1.63 × $10^{-5}$ for the fine mesh. Geometry response was used as the primary convergence basis because contact-edge singularities can affect global peak stress and strain. These mesh-sensitivity and energy-balance results further support the numerical suitability of the selected Yeoh input over the simulated deformation range.

### 2.3. Profile Extraction and Auxiliary Field Definitions

The simulated riblet-profile response was quantified from top-surface centerline profiles taken at the final forming state. The same forming-state profile was used for all cases, providing a consistent basis for comparing the temperature-series and *D*-series responses. The edge-plateau and valley ordinates are denoted as $y_{\mathrm{edge}}\left(D,T\right)$ and $y_{\mathrm{valley}}\left(D,T\right)$, respectively, and the formed riblet height is

(3) $$h_{f}\left(D,T\right)=y_{\mathrm{edge}}\left(D,T\right)-y_{\mathrm{valley}}\left(D,T\right)$$

The filling ratio is

(4) $$R_{f}\left(D,T\right)=\frac{h_{f}\left(D,T\right)}{h_{0}}\times 100\%$$

where $h_{0}$ = 100 μm.

The edge-to-valley extraction rule was applied consistently to simulated centerline profiles and laser-confocal riblet-depth measurements. The edge ordinate was taken from the local edge-plateau level, and the valley ordinate was taken from the local minimum within the riblet-valley window. This common rule defines $h_{f}$ and $R_{f}$ consistently for simulation and experiment, with $h_{0}=100\;\mu m$ used as the common nominal reference.

A sensitivity check showed that varying the edge-plateau window changed $R_{f}$ by less than 1 percentage point, while replacing the local minimum with a fitted valley ordinate changed $R_{f}$ by less than 3.7 percentage points; the monotonic *D*-series trend was preserved.

The auxiliary field quantities retained for A(D,T) are contact pressure, logarithmic strain, von Mises stress, and quasi-static solution status. Contact pressure describes contact localization, logarithmic strain describes the local strain response, and von Mises stress describes the stress-field response around the riblet-forming contact region. These fields complement the scalar geometry metrics $h_{f}$ and $R_{f}$ by providing local contact, strain, and stress information for process-window screening.

The same profile-height definitions can be applied to laser-confocal cross-sectional profiles. For a measured profile,

(5) $$h_{f,\exp}=y_{\mathrm{edge},\exp}-y_{\mathrm{valley},\exp}$$

and

(6) $$R_{f,\exp}=\frac{h_{f,\exp}}{h_{0}}\times 100\%$$

When a measured profile is paired with a simulated reference profile under a shared reference and extraction rule, the profile differences can be expressed as $\Delta h_{f}=h_{f,\exp}-h_{f,\mathrm{sim}}$ and $\Delta R_{f}=R_{f,\exp}-R_{f,\mathrm{sim}}$. These definitions establish the comparison protocol between simulation-derived profile responses and laser-confocal morphology measurements.

### 2.4. Experimental Setup and Morphology Characterization

**Figure 5:**
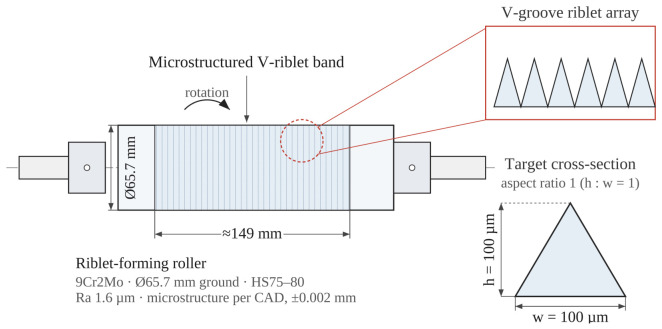
Schematic of the forming roller and its V-shaped forming features. The main view shows the roller dimensions and microstructured axial region; the enlarged views show the periodic forming-feature array and its nominal 100 μm × 100 μm cross-section.

R2R hot embossing experiments were conducted using a one-piece forming roller carrying periodic V-shaped forming ridges. The roller had a nominal working diameter of 65.7 mm and a microstructured axial region of approximately 149 mm. The forming ridges had a nominal height and width of 100 μm, corresponding to an aspect ratio of 1. The roller was precision-ground before microstructuring and was fabricated from 9Cr2Mo cold-work die steel. Its working surface was quench-hardened to HS75–80, with a hardened case depth greater than 6 mm and a surface roughness of Ra 1.6 μm. The forming features were machined according to the tool CAD model and controlled to a form tolerance of ±0.002 mm. Figure 5 shows the forming roller and the target V-shaped riblet geometry.

The large hardness contrast between the quench-hardened 9Cr2Mo forming roller and the soft hyperelastic FKM sheet supports treating roller wear as negligible over the duration of the present experiments, allowing the analysis to focus on the forming response and replicated morphology of the FKM sheet.

**Figure 6:**
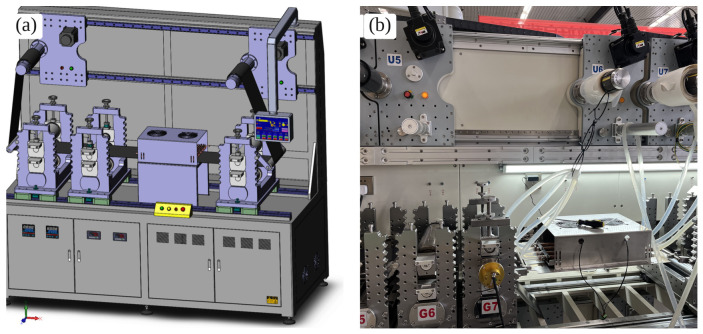
Roll-to-roll hot embossing apparatus used to prepare riblet-textured FKM sheets: (**a**) CAD layout; (**b**) assembled experimental apparatus.

The R2R hot embossing apparatus comprised the forming roller, support roller, strip-feeding, alignment and tensioning components, a heating unit, a cooling route, take-up components, and the associated temperature and motion controls. Figure 6a presents the CAD layout of these components, and Figure 6b shows their realization in the assembled experimental system. Together, the two panels connect the functional equipment layout to the apparatus used for the continuous feeding, embossing, cooling, and collection sequence.

During fabrication, the 1.0 mm-thick FKM strip was fed, aligned and preheated before entering the nip between the heated forming roller and support roller. The periodic tool profile was then embossed into the sheet, after which the textured strip exited the nip, passed through the cooling route, and was collected by the take-up module.

Four morphology-map conditions combined embossing temperatures of 120 and 160 °C with nominal nip-compression settings of 80 and 100 μm. A separate experimental *D*-series at 160 °C used nominal nip-compression levels of 60, 90, 100, and 120 μm. Preheating temperature, embossing temperature, web speed, cooling route, and sheet thickness were held constant across this series so that retained morphology could be compared as a function of nominal nip compression.

In the apparatus, *D* denotes the nominal commanded roller-spacing setting. Conversion to a run-specific effective nip-compression depth requires the zero-contact position, machine compliance, thermal expansion, and, when available, the measured nip force. The effective depth is expressed as

(7) $$D_{\mathrm{eff}}=u_{\mathrm{cmd}}-u_{0}-C_{m}F_{N}-\Delta u_{T},$$

where $u_{\mathrm{cmd}}$ is the commanded roller-spacing change, $u_{0}$ is the zero-contact reference, $C_{m}F_{N}$ accounts for machine-compliance deflection under the measured nip force $F_{N}$, and $\Delta u_{T}$ accounts for thermal expansion of the tooling stack. When nip force is recorded, the equivalent $D_{\mathrm{eff}}$ can also be obtained by matching the experimental line load $q_{N,\exp}$ to the FE line load $q_{N,\mathrm{FE}}\left(D\right)=F_{N,\mathrm{FE}}\left(D\right)/b_{\mathrm{FE}}$ and interpolating along the simulated reaction-force curve. Equation (7) therefore provides the calibration route between the nominal apparatus setting and the effective nip-compression depth.

Laser-confocal profilometry provides non-contact height-field measurement for riblet morphology and cross-sectional profile characterization [32,33]. An Olympus LEXT OLS system was used to measure the embossed riblet morphology, and the exported height maps were used for profile-based assessment. Profiles were extracted from central regions of the measured fields to avoid edge effects. Representative profile comparisons were based on averaged cross-sectional traces, whereas the *D*-series statistics were obtained from individual riblet measurements at each nominal nip setting.

For each nominal nip-compression level in the quantitative 160 °C comparison, 20 complete riblet cross-sections were analyzed. The experimental top width, $w_{t,\exp}$, was measured as the horizontal distance between the two upper edge points of each V-shaped riblet in the laser-confocal cross-sectional profile. The corresponding FE upper-edge width, $w_{t,\mathrm{sim}}$, was defined by the horizontal distance between the two rigid-tool shoulder positions that bound the V-shaped forming feature, located at x=−50μm and x=+50μm relative to the riblet center. Because the rigid tool underwent only vertical displacement, this upper-edge span remained 100 μm for all *D* conditions. The reported values are expressed as the mean ± sample standard deviation across the 20 riblet cross-sections analyzed for each condition. The resulting dispersion characterizes riblet-to-riblet morphological variation rather than independent process-to-process repeatability. A three-point moving average was used only to aid profile visualization and was excluded from all quantitative calculations.

## 3. Results

### 3.1. Temperature-Dependent Forming Response

**Figure 7:**
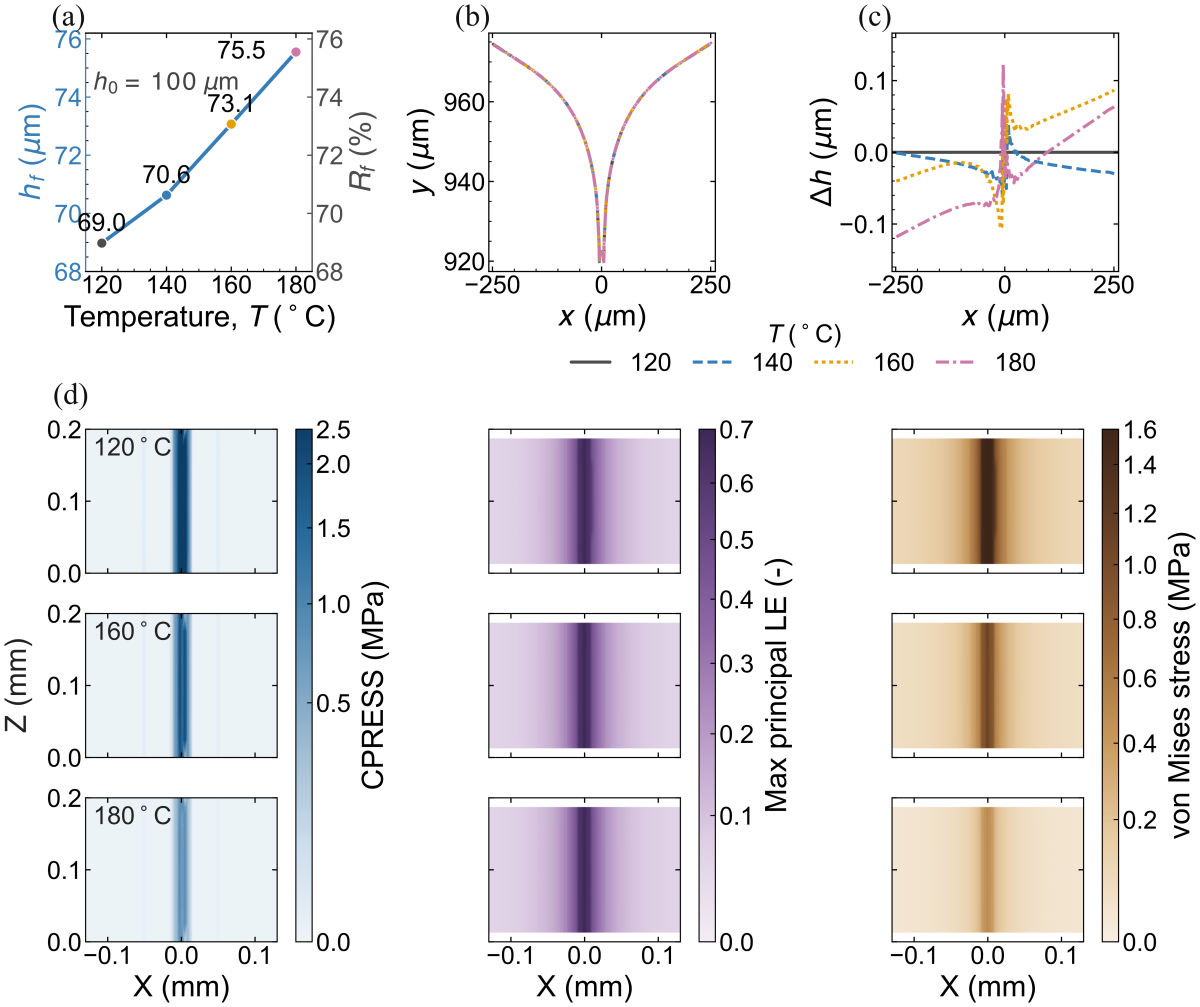
Temperature-series geometry response and auxiliary field maps. (**a**) Filling response; (**b**) profile overlay; (**c**) height-difference profile; (**d**) auxiliary field maps.

Figure 7 summarizes the temperature-dependent forming response over the evaluated 120–180 °C range. It combines the filling response, profile comparison, height-difference profile and auxiliary field maps used in the following analysis.

Within the equivalent hot-state representation, increasing embossing temperature from 120 to 180 °C raised the simulated filling ratio from 68.98% to 75.55% (Figure 7a). The 6.57 percentage-point rise indicates a modest temperature-assisted filling effect under the adopted material representation.

Within the equivalent hot-state coefficient representation, higher embossing temperature promotes the simulated riblet-profile filling in the evaluated cases [22,24,27,28,29]. The increase is consistent with lower effective deformation resistance at higher temperature, allowing the FKM sheet to fill the V-shaped riblet profile slightly more deeply under the same profile definition. Accordingly, the temperature-series result is interpreted as a qualitative process-screening trend obtained from the equivalent hot-state coefficient representation.

Although the filling ratio increased monotonically with temperature, the physical-scale profile overlay shows that the formed profiles remained nearly coincident (Figure 7b). The height-difference profile relative to 120 °C localizes this small temperature-induced change near the riblet valley and adjacent sidewalls (Figure 7c), whereas the scalar filling ratio remains the primary measure of temperature-assisted filling.

The auxiliary field maps in Figure 7d extend the temperature-series result from scalar geometry response to local field distribution. At the fixed nip-compression depth used for the temperature series, D=80μm, the extracted peak-field values show that CPRESS and von Mises peaks decrease as embossing temperature increases from 120 to 180 °C, and the maximum-principal logarithmic-strain peak remains nearly unchanged. The concurrent drop in contact pressure and von Mises stress is consistent with a softened sheet filling the same cavity under lower resistance, while the peak maximum-principal logarithmic strain stayed nearly constant.

### 3.2. Nip-Compression-Depth-Dependent Forming Response

**Figure 8:**
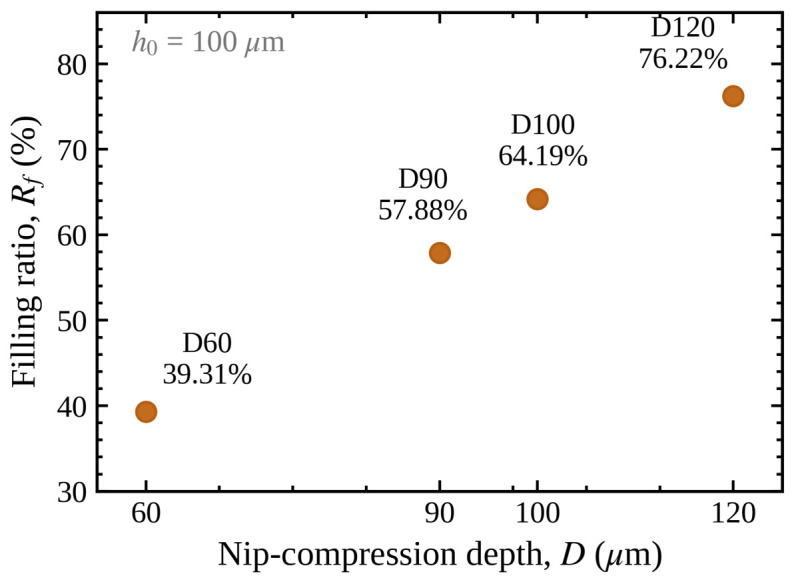
*D*-series geometry response under nip-compression-depth variation. Markers denote the simulation conditions D=60, 90, 100, and 120μm used for experimental comparison.

The prescribed nip-compression depth *D* is the second main process variable and provides the more direct geometric control of riblet-profile filling. The *D*-series simulations were evaluated at D=60, 90, 100, and 120μm under the fixed thermal context of 140 °C preheating and 160 °C embossing. Figure 8 relates these prescribed depths to the formed height and filling ratio calculated using $h_{0}=100\;\mu$m.

Across the sampled *D*-series, the filling ratio rises from 39.31% at D=60μm to 76.22% at D=120μm. The 36.91 percentage-point range is much larger than the temperature-series change over 120–180 °C, indicating that the prescribed nip-compression depth provides the strongest geometric control of riblet-profile filling in the examined simulation set. The response at D=100μm, with $R_{f}=64.19\%$, provides a moderate-filling reference, whereas D=120μm, with $R_{f}=76.22\%$, gives the highest filling in the reported *D*-series and provides the high-filling reference for field and morphology assessment.

**Figure 9:**
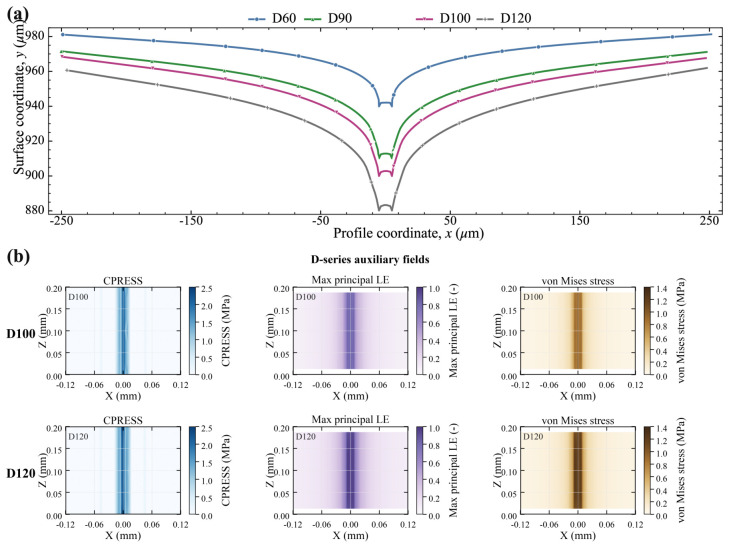
D-series profile response and auxiliary field maps. (**a**) Centerline profiles; (**b**) auxiliary field maps.

The centerline profiles deepen progressively as *D* increases from 60 to 120 μm (Figure 9a), mirroring the monotonic filling-ratio rise in Figure 8 and showing how nip compression reshapes the riblet cross-section together with the scalar filling response.

The auxiliary field maps in Figure 9b compare D=100μm and D=120μm under the same 140 °C preheating and 160 °C embossing simulation context. Over this increase in *D*, the peak contact pressure rises from about 1.69 to 2.28 MPa, the peak von Mises stress rises from about 1.03 to 1.30 MPa, and the peak maximum-principal logarithmic strain rises from about 0.83 to 0.97. Thus, the higher-filling response at D=120μm also carries higher auxiliary field indicators in the displayed comparison. For process screening, this result shows that *D* improves filling efficiently and that the accompanying CPRESS, strain, and stress fields help identify high-filling conditions for morphology assessment and further process refinement [19,20,27,28,29].

### 3.3. Field-Informed Screening Framework Derived from the Simulation Results

The temperature-series and *D*-series results are integrated into a field-informed screening framework. The geometry component evaluates formed height, filling ratio, and profile response, whereas the auxiliary-field component includes contact pressure, logarithmic strain, von Mises stress, and the quasi-static energy check. This two-component structure integrates geometry and auxiliary-field responses into a process-window framework for morphology-guided experimental assessment.

The geometry response is defined as

(8) $$G\left(D,T\right)=\{h_{f}\left(D,T\right),\;R_{f}\left(D,T\right),\;\mathrm{profile}\;\mathrm{response}\left(D,T\right)\}$$

The auxiliary-field response is defined as

(9) $$\begin{array}{c} A(D,T)=\{\mathrm{CPRESS},\;\mathrm{LE},\\ \;\mathrm{von}\;\mathrm{Mises}\;\mathrm{stress},\;\mathrm{quasi}\text{-}\mathrm{static}\;\mathrm{check}\}. \end{array}$$

Here, A(D,T) denotes the auxiliary-field and quasi-static solution information. The selected process set is expressed as

(10) $$\begin{array}{cc} \Omega_{\mathrm{process}}=\{\left(D,T\right)|\; & G(D,T)\;\mathrm{supports}\;\mathrm{profile}\;\mathrm{filling},\\ \; & A(D,T)\;\mathrm{provides}\;\mathrm{auxiliary}\text{-}\mathrm{field}\;\mathrm{context}\}. \end{array}$$

**Figure 10:**
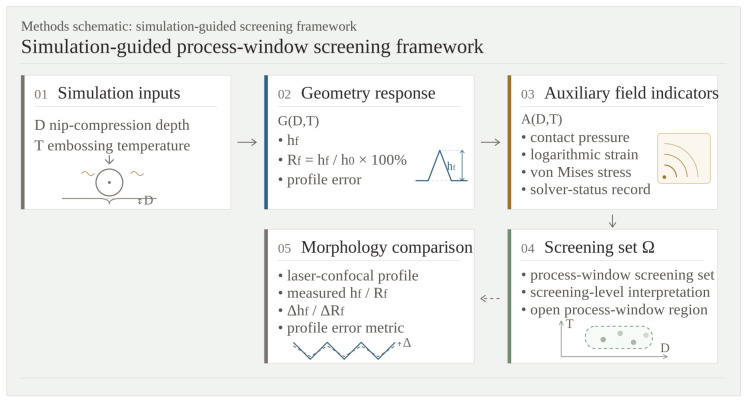
Simulation-guided process-window screening framework.

Figure 10 summarizes the framework by relating the simulation inputs, geometry response G(D,T), auxiliary-field response A(D,T), selected process set $\Omega_{\mathrm{process}}$, and experimental morphology assessment. The schematic shows how the temperature response, *D*-series response, and experimental assessment are combined within the integrated numerical–experimental framework, with auxiliary fields serving as defect-risk indicators alongside the geometry response [27,28,29,33].

**Figure 11:**
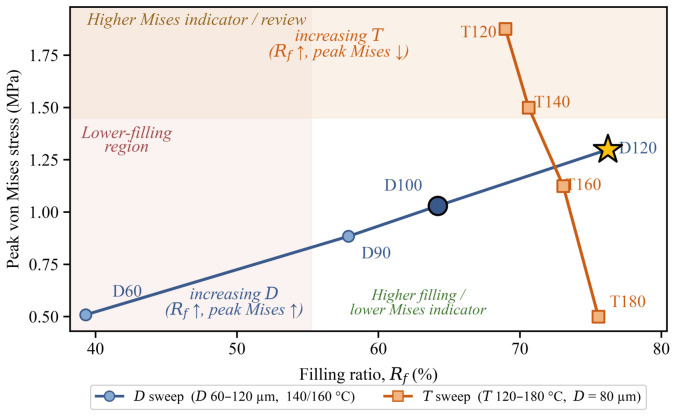
Geometry-field screening map. Blue circles and orange squares denote the *D* and *T* sweeps, respectively; arrows indicate increasing *D* or *T*, and the star marks the D=120μm high-filling reference.

Plotting each case in the ($R_{f}$, peak von Mises) plane separates the two process levers (Figure 11): deeper nip compression increases filling at the cost of rising local stress, whereas higher temperature increases filling while reducing the plotted stress indicator.

Near the highest filling range, D=120μm reaches a filling ratio near 76% with a peak von Mises stress of about 1.3 MPa. The temperature-series response at T=180 °C reaches a similar filling level with a lower plotted peak von Mises-stress indicator of about 0.5 MPa. The map therefore provides a compact comparison of how temperature and nip-compression depth reach similar filling levels through different field-response paths. The map supports process-condition prioritization and identifies conditions for morphology assessment; quantitative acceptance limits require expanded process maps.

Table 5 summarizes the auxiliary field outputs used for the relative process-condition comparison. In the *D*-series field maps, the peak CPRESS values at D=60, 100, and 120μm are approximately 1.05, 1.69, and 2.28 MPa, respectively, while the corresponding peak von Mises values are approximately 0.51, 1.03, and 1.30 MPa. At D=120μm, the peak maximum-principal LE approaches 0.97 and is treated as a high-strain localization condition for review. In the temperature-series maps, increasing temperature reduces the plotted CPRESS and von Mises peaks, with the peak von Mises indicator decreasing from about 1.88 MPa at 120 °C to about 0.50 MPa at 180 °C. The field indicators distinguish high-filling conditions from those requiring defect-risk review and provide a screening basis for process-condition selection.

The indicators in Table 5 serve as relative process-condition comparison metrics, while material-specific thresholds can be established through dedicated defect validation on riblet-textured FKM sheets.

### 3.4. Process-Window Screening and Experimental Target

Combining the temperature-series and *D*-series results identifies a candidate process region for experimental assessment. Based on the simulation screening, 160 °C was selected as the representative thermal condition for the experimental *D*-series because it provided improved profile filling relative to the lower-temperature cases together with reduced auxiliary field indicators. This temperature therefore served as the thermal context for the subsequent nip-compression series. The *D*-series then identifies the moderate-to-high-filling range between D=100μm and D=120μm as the most relevant geometry interval for follow-up assessment. The response at D=100μm provides a reference filling level of about 64%, whereas D=120μm reaches the highest filling level of about 76% in the reported *D*-series.

**Figure 12:**
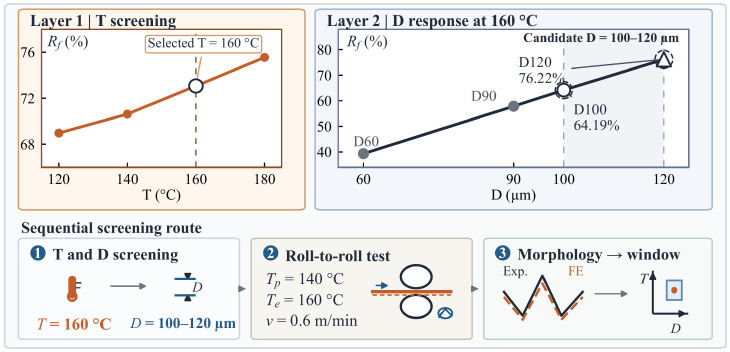
Temperature-first process-screening route. The orange and blue lines show the temperature- and nip-compression-depth-dependent filling responses, respectively. Dashed vertical lines mark the selected T=160 °C condition and the candidate D=100–120μm interval, while solid arrows show the sequential route from numerical screening to R2R testing and morphology-based window assessment.

Figure 12 summarizes the temperature-first screening sequence. The embossing-temperature context is first identified from the temperature-series response, the prescribed nip-compression depth is then used to control filling more directly, and the resulting candidate process region is connected to experimental morphology assessment. The representative R2R conditions were selected with reference to this simulation-guided screening region, and the retained-profile trend was evaluated at nominal settings of D=60, 90, 100, and 120μm. Relative to a full 4×5 temperature–*D* matrix containing 20 morphology conditions, the screening strategy reduced the initial experimental assessment to four morphology-map conditions and a four-condition quantitative *D*-series. Because the 160 °C, D=100μm condition was included in both groups, these experiments comprised eight datasets spanning seven unique *T*–*D* combinations. This estimate is specific to the temperature, nip-setting, and repetition levels defined in this study, but it illustrates the role of the screening strategy in narrowing the experimental morphology-assessment space.

## 4. Experimental Morphology Assessment for Process-Window Screening

### 4.1. Laser-Confocal Morphology of the Embossed Riblets

**Figure 13:**
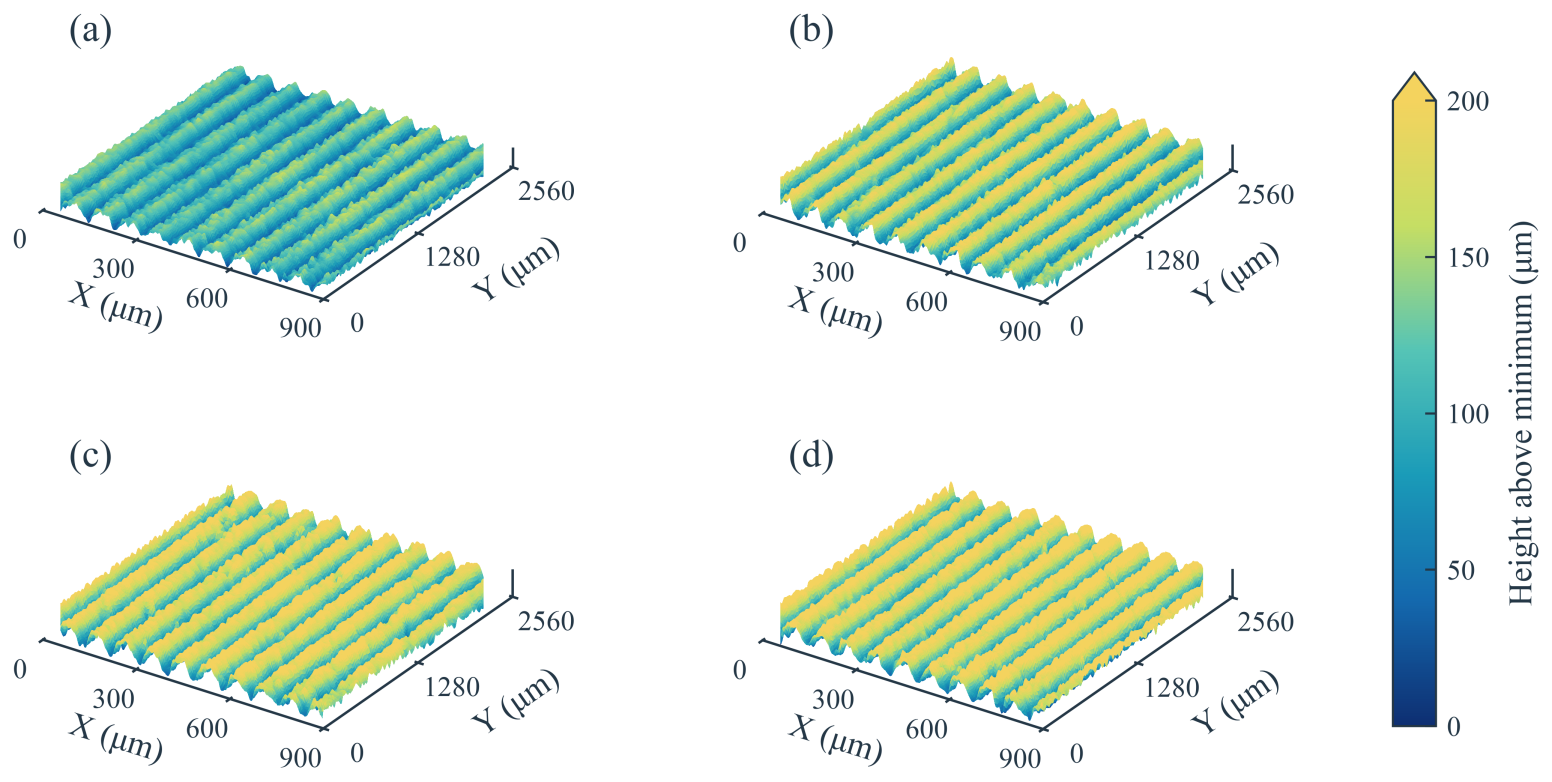
Laser-confocal morphology maps. (**a**) 120 °C, 80 μm; (**b**) 120 °C, 100 μm; (**c**) 160 °C, 80 μm; (**d**) 160 °C, 100 μm.

The R2R hot embossing assessment evaluated the riblet morphology across four representative temperature–nip combinations used to visualize the experimental screening trends. Figure 13a,b shows the laser-confocal morphology obtained at 120 °C with nominal nip settings of 80 and 100 μm, respectively, whereas Figure 13c,d shows the corresponding two nip settings at 160 °C. The panel arrangement separates the temperature comparison by row and the nip-setting comparison within each row.

Continuous, aligned V-shaped riblet arrays are observed in all four measured fields, and the replicated ridge–valley features remain periodic after embossing. Panels (a)–(d) resolve regularly spaced parallel ridges with clearly defined crests and valley floors across the scanned area, while the ridge-to-valley height contrast varies slightly among the four temperature–nip combinations. The maps therefore document texture transfer across the evaluated morphology-map conditions and provide the surface-height fields used in the subsequent profile-based comparison.

### 4.2. Measured Profile and Simulation Reference

**Figure 14:**
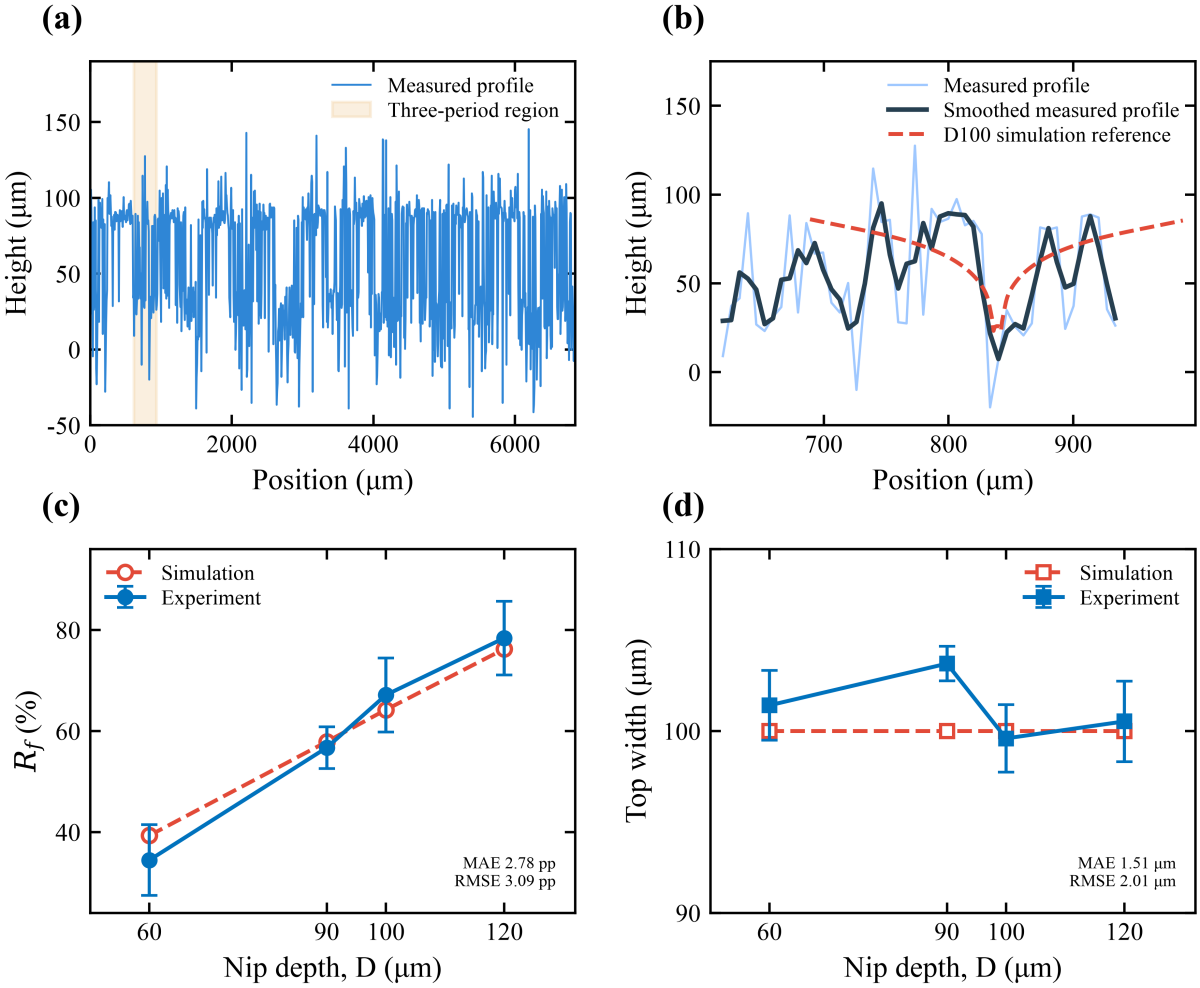
Measured and simulated morphology statistics. (**a**) Measured centerline profile and selected three-period region; (**b**) measured profile, smoothed measured profile, and simulation reference at D=100μm; (**c**) filling ratio; (**d**) top width.

Figure 14 connects the measured cross-sectional morphology to the simulated references for the 160 °C *D*-series. Panel (a) shows the complete laser-confocal centerline trace and identifies the three-period region selected for local profile visualization. Panel (b) compares that measured region and its display-only three-point moving average with the simulated profile at D=100μm. Panels (c) and (d) then compare the measured group means and standard deviations with the corresponding simulations for filling ratio and top width, respectively.

The measured trace describes the riblet shape retained on the FKM sheet after R2R hot embossing, whereas the simulated profile in Figure 14b provides the forming-stage reference for the corresponding nominal *D* setting. The dark curve is a three-point moving-average representation of the measured trace and is used only to make the local three-period profile easier to compare visually with the simulation. The filling ratio in Figure 14c was calculated from the measured riblet depth using $h_{0}=100\;\mu$m, and the top width in Figure 14d was measured from the same riblet cross-sections. The close group-level trends in panels (c) and (d), quantified by their MAE and RMSE values, connect the forming-stage predictions to the retained riblet geometry.

Across nominal settings from D=60μm to D=120μm, the measured retained filling ratio increased with nip compression and followed the simulated *D*-series trend (Table 6).

The condition-wise filling-ratio deviation was calculated as

(11) $$\Delta R_{f}=R_{f,\exp}-R_{f,\mathrm{sim}},$$

where $\Delta R_{f}$ is reported in percentage points (pp).

The four filling-ratio groups remained within 4.86 percentage points of the corresponding simulated values, giving MAE = 2.78 percentage points and RMSE = 3.09 percentage points. The top-width comparison gave MAE = 1.51 μm and RMSE = 2.01 μm. At D=100μm and D=120μm, the retained experimental mean is slightly higher than the forming-stage simulation value at the corresponding nominal setting. Run-specific correspondence between the experimental setting and effective nip-compression depth requires the calibration in Equation (7); the small positive deviations at the two higher settings are consistent with an uncalibrated offset in that correspondence. Overall, the simulated *D*-series response provides a forming-stage guide for retained-morphology assessment.

Together, the laser-confocal maps and *D*-series morphology metrics provide the experimental assessment for the proposed screening framework. The simulations identify how embossing temperature and nip-compression depth influence profile filling and auxiliary field response, and the measured morphology shows that representative process conditions can produce periodic riblet structures on the FKM sheet. This agreement supports expanded *T*–*D* process-window mapping based on combined simulation and morphology measurements.

## 5. Discussion

### 5.1. Effects of Temperature and Nip-Compression Depth on Riblet-Profile Filling

The temperature-series and *D*-series results reflect two distinct contributions to riblet-profile filling. Increasing the embossing temperature from 120 to 180 °C raised the simulated filling ratio from approximately 69% to 76%. Increasing the imposed nip-compression depth *D* from 60 to 120 μm raised it from approximately 39% to 76%. The comparison indicates that temperature contributes through the temperature-dependent material scaling, while the imposed nip-compression depth acts as the more direct geometric control over riblet-profile filling. The temperature trend is consistent with a reduction in effective deformation resistance at higher temperature, allowing the sheet to enter the V-shaped riblet-forming cavity more deeply under the same profile definition.

At D=120μm, the simulated formed profile reached $h_{f}=76.22\;\mu$m for $h_{0}=100\;\mu$m, corresponding to $R_{f}=76.22\%$. For a hyperelastic sheet, forming-stage filling and post-embossing morphology are distinct quantities. The simulation therefore provides the forming-stage filling indicator, whereas laser-confocal measurements quantify the retained profile after R2R embossing and cooling. The comparison between the forming-stage simulation and retained morphology links the predicted filling response to the experimentally produced riblet geometry [23,24,34,35].

The response at D=120μm represents a high-filling, partially filled forming-stage profile for the evaluated hyperelastic FKM sheet. This result links increased nip compression with improved forming-stage filling. In the 160 °C *D*-series measurements, the retained filling ratio increased with the nominal nip-compression setting, and the response at D=120μm remained close to its simulated reference. Taken together, the forming-stage filling, retained morphology, and local field response support the candidate process region used for morphology assessment.

Geometry response and auxiliary field indicators are used together for process-window screening. The contact-pressure, logarithmic-strain, and von Mises stress fields identify where deeper compression may produce local concentration near the riblet profile. The indicators in Table 5 link these fields to possible cracking or tearing, wrinkling or folding, contact-induced surface damage, and interfacial separation risk. High-filling *D*-series conditions such as D=120μm are therefore prioritized for further defect-risk assessment based on both profile filling and auxiliary field indicators. Together, the results support a process-screening interpretation: temperature raises the tendency for filling, nip-compression depth controls the extent of mechanical filling more directly, and the auxiliary fields provide defect-risk indicators for process-window refinement.

### 5.2. From Simulated Profiles to Measured Morphology

For process selection, the retained riblet morphology after embossing and cooling is the relevant manufacturing output. The simulated $h_{f}$ and $R_{f}$ trends provide forming-stage guidance, whereas laser-confocal profilometry quantifies the retained riblet cross-section, including its geometric fidelity, peak-to-valley features, and local profile variation.

The *D*-series comparison provides a quantitative link between simulation screening and laser-confocal measurements. At the group level, the measured filling ratio and top width remained close to their corresponding forming-stage references, with filling-ratio RMSE = 3.09 percentage points and top-width RMSE = 2.01 μm. The condition at D=120μm showed the highest retained filling, consistent with the increasing forming-stage trend predicted by the *D*-series simulation. Because the experimental values are nominal nip-compression settings, run-specific quantitative correspondence requires the calibration of $D_{\mathrm{eff}}$. The observed agreement supports the simulated *D*-series trend as a forming-stage guide and the measured retained morphology as the basis for process-window refinement.

Beyond the condition-level numerical–experimental agreement, the central manufacturing result is the continuous formation and retention of microscale V-shaped riblet textures on a hyperelastic FKM sheet. The simulation identified the forming conditions for experimental assessment, and the R2R experiments showed that the periodic microstructure could be transferred to and retained by the flexible elastomeric carrier after R2R hot embossing. This flexible textured-sheet format provides a practical manufacturing basis for potential integration with curved, non-planar, or geometrically complex substrates and thus for the broader deployment of such microstructured surfaces.

### 5.3. Limitations and Outlook

The integrated numerical–experimental framework establishes a basis for selecting R2R hot embossing conditions. Within the equivalent hot-state material representation, the temperature-series results describe process-screening trends for the evaluated FKM sheet. The simulations describe the forming-stage profile, and laser-confocal measurements quantify the retained morphology after embossing and cooling. Conversion of the experimental nominal nip-compression settings into run-specific effective depths requires dedicated calibration. Further development of the framework should include temperature-dependent constitutive calibration, refined contact calibration, and expanded coupled *T*–*D* process mapping.

## 6. Conclusions

1. A simulation-guided process-window screening method was established for R2R hot embossing of 100 μm-scale V-shaped riblets on hyperelastic FKM sheets. The method integrates a Yeoh-based large-deformation contact model with laser-confocal morphology assessment to connect forming-stage profile prediction with retained riblet morphology.
2. The simulations showed that prescribed nip-compression depth provided the stronger geometric control of riblet-profile filling within the evaluated process range. The *D*-series filling ratio increased from about 39% at D=60μm to about 76% at D=120μm, identifying D=100–120 μm as the main morphology-assessment interval.
3. R2R hot embossing experiments showed that periodic V-shaped riblet structures could be retained on the FKM sheet under the selected process conditions. The 160 °C *D*-series followed the simulated filling trend, with filling-ratio RMSE of 3.09 percentage points and top-width RMSE of 2.01 μm.
4. The integrated numerical–experimental framework provides a practical route for continuously forming and retaining microscale V-shaped riblets on hyperelastic FKM sheets. By linking material characterization, forming simulation, and roll-to-roll fabrication, the approach offers a process-oriented strategy for transferring microtextures from the forming roller to a flexible elastomeric carrier. The conformable microstructured-sheet format is potentially compatible with curved, non-planar and geometrically complex substrates and may therefore extend the deployment of such biomimetic microstructures to broader engineering applications.

## Figures and Tables

**Table 1:** Yeoh model parameters used in the Abaqus simulations.

Parameter	Value
$C_{10}$	0.784250 MPa
$C_{20}$	0.004795 MPa
$C_{30}$	−0.000274 MPa

**Table 2:** Comparison of fitted hyperelastic constitutive models for the room-temperature tensile response.

Metric	Yeoh	Ogden	Ogden − Yeoh
R^2^	0.996233	0.997080	+0.085%
MAE (kPa)	39.32	33.15	−6.17
RMSE (kPa)	50.65	44.59	−6.06
maximum absolute error (kPa)	294.69	328.92	+34.23
number of fitted parameters	3	4	-
negative fitted parameters	Yes ($C_{30}$)	Yes ($\mu_{2}$, $\alpha_{2}$)	-

Note: Differences were calculated as Ogden minus Yeoh. The $R^{2}$ difference is reported as a relative percentage, whereas the MAE, RMSE, and maximum-absolute-error differences are reported as absolute differences in kPa.

**Table 3:** Local field-sensitivity response at D=80μm under 130 °C preheating, 150 °C embossing, s(150 °C) = 0.18, and 0.6 m/min web speed.

Friction Coefficient	Max|LE|	ALLKE/ALLIE
0	1.181	6.80 × 10^−4^
0.05	1.177	1.02 × 10^−3^
0.10	1.215	1.11 × 10^−3^

**Table 4:** Equivalent hot-state Yeoh coefficients used in the screening simulations.

*T* (°C)	s(T)	$C_{10}\left(T\right)$ (MPa)	$C_{20}\left(T\right)$ (MPa)	$C_{30}\left(T\right)$ (MPa)
120	0.30	0.235275	0.0014385	−8.220 × 10^−5^
140	0.24	0.188220	0.0011508	−6.576 × 10^−5^
150	0.18	0.141165	0.0008631	−4.932 × 10^−5^
160	0.12	0.094110	0.0005754	−3.288 × 10^−5^
180	0.08	0.062740	0.0003836	−2.192 × 10^−5^

**Table 5:** Auxiliary field indicators used for relative process-condition comparison.

Defect ModeConsidered	Main Indicator	Comparison Indicator
Surface cracking or tearing	Maximum-principal logarithmic strain and von Mises stress	Lower maximum-principal LE and von Mises stress; 2.92 MPa tensile-response upper reference.
Wrinkling or local folding	Profile instability combined with strain localization	Profile opening and localized LE distribution.
Contact-induced surface damage or sticking	CPRESS distribution at the tool–sheet interface	Peak CPRESS relative to the 2.5 MPa screening reference, reviewed with local stress and strain.
Interfacial separation or delamination risk	Combined stress and contact-pressure localization	Combined local stress and CPRESS concentration.

**Table 6:** Condition-wise comparison between forming-stage simulation and retained experimental morphology in the 160 °C *D*-series.

Nominal *D* Setting (μm)	Simulated $R_{f}$, Forming Stage (%)	Measured Retained $R_{f}$ (%)	$\Delta R_{f}$ (pp)
60	39.31	34.45 ± 7.01	−4.86
90	57.88	56.71 ± 4.13	−1.17
100	64.19	67.12 ± 7.33	+2.93
120	76.22	78.38 ± 7.29	+2.16

## Data Availability

The data that support the findings of this study are available from the corresponding author upon reasonable request.
